# Pharmacometric approach to assist dosage regimen design in neonates undergoing therapeutic hypothermia

**DOI:** 10.1038/s41390-021-01714-0

**Published:** 2021-09-07

**Authors:** Saikumar Matcha, Elstin Anbu Raj, Ramya Mahadevan, Arun Prasath Raju, V Rajesh, Leslie Edward Lewis, Surulivelrajan Mallayasamy

**Affiliations:** 1 Department of Pharmacy Practice, Manipal College of Pharmaceutical Sciences, Manipal Academy of Higher Education, Manipal, Udupi, Karnataka India; 2 PUMAS-AI, Inc., Baltimore, MD USA; 3 Department of Paediatrics, Kasturba Medical College, Manipal Academy of Higher Education, Manipal, Udupi, Karnataka India

## Abstract

**Background:**

Therapeutic hypothermia (TH) is the treatment of choice for neonates diagnosed with perinatal asphyxia (PA). Dosing recommendations of various therapeutic agents including antimicrobials were not specifically available for the neonates undergoing TH.

**Methods:**

A systematic search methodology was used to identify pharmacokinetic (PK) studies of antimicrobials during TH. Antimicrobials with multiple PK studies were identified to create a generalizable PK model. Pharmacometric simulations were performed using the PUMAS software platform to reproduce the results of published studies. A suitable model that could reproduce the results of all other published studies was identified. With the help of a generalizable model, an optimal dosage regimen was designed considering the important covariates of the identified model.

**Results:**

With the systematic search, only gentamicin had multiple PK reports during TH. A generalizable model was identified and the model predictions could match the reported/observed concentrations of publications. Birth weight and serum creatinine were the significant covariates influencing the PK of gentamicin in neonates. A dosage nomogram was designed using pharmacometric simulations to maintain gentamicin concentrations below 10 μg/mL at peak and below 2 μg/mL at trough.

**Conclusions:**

A generalizable PK model for gentamicin during TH in neonates was identified. Using the model, a dosing nomogram for gentamicin was designed.

**Impact:**

- Dosing guidelines for antimicrobials during TH in neonates is lacking.
- This is the first study to identify the generalizable model for gentamicin during TH in neonates.
- Nomogram, proposed in the study, will aid the clinicians to individualize gentamicin dosing regimen for neonates considering the birth weight and serum creatinine.

## Introduction

Perinatal asphyxia (PA) is a condition in which the newborn is deprived of oxygen during the birth process.^1^ PA affects four million newborns each year, resulting in one million neonatal deaths worldwide.^1^ Neonates with PA are more likely to develop encephalopathy and acute renal injury, as well as other infections, because of their extended stay in the hospital.^2,3^ For newborns with PA, therapeutic hypothermia (TH) is the recommended treatment. TH is a 72-h process in which the core body temperature is maintained at 34–33 °C, followed by a 6-h rewarming phase to normal body temperature.^4–6^ TH can lead to a number of physiological abnormalities affecting various organs including kidneys, leading to the accumulation of toxins and drugs.^7–9^ PA is managed with TH, along with antiepileptics, antimicrobials, and other medications, depending on the complications.^10^

Antimicrobial prescribing guidelines for newborns are available; however, its use during TH is still a challenge. One of the primary reasons for the complexity of dosing guidelines appears to be the lack of pharmacokinetic (PK) data during TH. To prevent subtherapeutic or toxic effects, antimicrobials should be maintained within the therapeutic window.

Population PKs (PopPKs) is a pharmacometric discipline that facilitates in identifying and accounting for sources of variability.^11^ A pharmacometric approach can be used to simulate the time vs. concentration profile of a drug for an individual, taking into account the significant covariates that were found to influence the PK parameters.^12^ Antimicrobial dosing regimens for asphyxiated infants undergoing TH are not available. Due to the reduced clearance (CL) of several antimicrobials during TH, the standard dosing regimen used in neonates may result in toxic concentrations. The aim of this study was to conduct a thorough analysis of the PK of various antimicrobials prescribed during TH in newborns diagnosed with PA and to use meta-model approach and pharmacometric simulations to identify generalizable models. The dosage recommendation for this population will be provided based on the identified generalizable model

## Methods

We used previously reported PopPK models in neonates to generate virtual participants for use in the simulations in this investigation. No institutional review board approval was required for this study as no patient data were used. The species used in the study are the virtual neonatal population.

### Systematic review

Electronic databases such as Scopus, PubMed, Medline, and CINAHL were used to conduct a systematic search. The following keywords: “infant*” OR “neonate*” OR “newborn*”, “asphyxia neonatorum*” OR “birth asphyxia*” OR “Perinatal asphyxia*” OR “hypoxic ischemic encephalopathy”, “therapeutic hypothermia” OR “induced hypothermia” OR “controlled hypothermia” OR “cooling therapy”, “Anti-Bacterial agent*” OR “Anti-Infective Agent*” has been used to build the search strategy across the databases The studies that were included were completed before June 2020. The entire search approach is described in Table 1. PRISMA rules were followed when conducting the search.

**Table 1:** Demographic details and results of studies.

Study ID	Drug name	Gestational age		Body weight		CL		*V*_C_/T *V*_d_		Outcome
		TH	NT	TH	NT	TH	NT	TH	NT	
Cristea, 2017	Amikacin	38 (35–41)	31^a^ (24–43)	3.18 (1.91–4.8)	1.53^a^ (0.38–4.65)	0.0297 L/h/kg^b^	0.0495 L/h/kg	0.832^b^/1.664^b^ L/1.75 kg	0.832/1.664 L/1.75 kg	Clearance reduced by 41%
Bijleveld, 2017	Amoxicillin	40	^c^	3.34	^c^	0.26 L/h/3 kg	0.41 L/h/3 kg	0.34/0.68 L/kg	0.34^b^/0.68^b^ L/kg	CL increased to 0.41 L/h at PNA (23%) 5 days, temp. (27%) 37 °C
Cies, 2017	Ampicillin	39 (36–41)	34.9^a^	3.2 (2.4–4.9)	2.5^a^	0.0258 L/h/kg	0.084 L/h/kg^d^	0.35/0.52 L/kg	−/0.4^d^ L/kg	Reduced CL and increased Vd during TH
Cies, 2018	Gentamicin	39.2 ± 1.5	40^a^	3.38 ± 0.46	3.4^a^	0.132 L/h/kg	0.06 L/h/kg^d^	0.44/0.96 L/kg	0.45/0.96^d^ L/kg	Increased CL with TH
Bijleveld, 2016	Gentamicin	40 (36–42)	^c^	3.4 (2.09–5.07)	^c^	0.06 L//h/kg	0.077 l/kg/h^b^	0.46/0.89 L/kg	0.46/0.89^b^ L/kg	CL reduced by 29%
Ting, 2014	Gentamicin	39 (38, 40)	39 (36, 41)	3.3 (2.9–3.6)	3.35 (2.6–3.5)	0.033 L/h/kg	0.051 L/h/kg	0.41 L/kg	0.45 L/kg	Reduced CL
Mark, 2013	Gentamicin	38.4 ± 1.4	39.3 ± 1.7	2.93 ± 0.6	3.27 ± 0.53	0.04 L/h/kg	0.05 L/h/kg	0.46 L/kg	0.46 L/kg	CL reduced by 25.5%
Frymoyer, 2013a	Gentamicin	40.0 (37.6–40.7) (IQR)	33.24^a^	3.32 (2.97–3.50) (IQR)	1.93^a^	0.111 L/h/3.3 kg	0.048–0.072 L/h/kg^d^	0.566 L/3.3 kg	0.47 L/kg^d^	CL reduced by 25–50% when compared with previous reports of NT neonates
Frymoyer, 2013b	Gentamicin	39.3 ± 1.9/40.2 ± 1.1	^c^	3.26 ± 0.58/3.45 ± 0.57	^c^	1.17 L/h/70 kg	NA	NA	NA	NA
Liu, 2009	Gentamicin	39.5 ± 1.6	39.3 ± 1.3	3.4 ± 0.72	3.51 ± 0.58	NA	NA	NA	NA	No effect of TH on mean concentrations and trough levels

*TH* therapeutic hypothermia, *NT* normothermia, *CL* clearance, *V*_*C*_ volume of distribution of central compartment, *T V*_*d*_ total volume of distribution, *NA* data not available from reported studies.

^a^Compared with other study data as a reference.

^b^Estimated from study results.

^c^The study is neither two arm nor compared with other studies by the author.

^d^Compared with other published data by the author.

The screening was performed simultaneously by two reviewers (S.M. and E.A.R.) based on the inclusion criteria. To include the relevant publications, the abstracts were screened first, followed by full-text screening. For any further inclusion, the references of the included articles were also reviewed. Any disagreements that arose during the process were resolved through discussion. Two reviewers worked on data extraction concurrently and independently, using a predesigned data form to capture appropriate data pertinent to the review. The final study included PK and PopPK, which monitored primary PK parameters such as CL, volume of distribution (*V*_d_), and additional parameters such as elimination rate constant (*K*_e_), and half-life (*T*_1/2_). In the instance where two reviewers disagreed, a third reviewer (S.R.M.) was contacted to provide clarification. The evaluation includes prospective and retrospective, interventional and observational studies that investigated the PK of antimicrobial agents in neonates during TH. Nonhuman studies and non-English language studies were excluded.

### Pharmacometric simulations for identifying a generalizable model

A minimum of two PK studies were required for any drug to be used in the derivation of a generalizable model. The analysis includes PK studies of antimicrobial drugs that met the inclusion criteria. The investigations collected all available demographics and data, including peak concentration (*C*_max_), trough concentration (*C*_min_), and other PK parameters. PopPK studies were discovered among the studies included in the systematic review. Using the mean and median demographic data from the individual research, a typical subject representing that study’s population was created. For that population, the reported average dose was used as the normal dose. The characteristics of the PopPK models from selected studies were used to generate antimicrobial agent PK profiles. The replicated model was validated by simulating peak and trough concentrations from each study.

A PopPK model was chosen as a reference model, and simulations were performed using demographic data from the other studies to compare peak and trough values reported in the other studies. This process was repeated until all of the reported studies were compared to the published models. As a generalizable model, a definite model was chosen that could match the reported values of all or the majority of the studies.

Following the selection of a generalized model, pharmacometric simulations with relevant covariates were performed. All of the studies provided a set of covariate values that were tabulated. Using pharmacometric simulations, the optimal dosage regimen for individuals representing these tabulated covariate values was screened. Based on these simulations, an optimal dosage regimen for all individuals within the range of covariates was provided in the table. The Julia computing language’s PUMAS package version 1.1.0 was used to perform all of the simulations.^13^

## Results

A total of ten studies met the inclusion criteria. The use of gentamicin, amikacin, amoxicillin, and ampicillin for newborns during TH has been studied. Demographic details such as gestational age (GA), birth weight (BWT), and PK parameters like CL and *V*_d_ during TH from various studies are presented in Table 1. The PopPK approach was utilized to estimate PK parameters for the studies, which reported a PopPK model. PopPK modeling was performed in these studies using software packages such as NONMEM and nonparametric pharmacometric modeling and simulation package in R. Effect of various covariates on parameters and interindividual variability/between-subject variability of parameters was also reported in these studies. In these studies, the model was qualified using the bootstrap resampling process and visual prediction checks. The impact of TH on CL, *V*_d_ and the outcomes of the study are shown in Table 1. Since there were seven PK trials to compare, gentamicin models were taken further to identify a generalizable model. Other drugs were left out of this analysis because they only had one study.

When demographic and dosing regimen data were used for simulation, the Frymoyer model was identified as a generalizable model since it was able to match the reported concentrations (peak and trough) of other studies. Frymoyer et al. recommended an intravenous infusion of 4–5 mg/kg every 36 h to maintain gentamicin concentrations in the therapeutic range (peak around 10 mg/dL, trough below 2 mg/dL). The Frymoyer model is a one-compartment, first-order elimination model. BWT and serum creatinine (SCr) were identified as influential covariates on CL.^14^ Based on the available studies, a BWT of 2.5–4 kg and an SCr of 0.3–1.5 mg/dL were chosen as a range for pharmacometric simulations. Peak and trough concentrations simulated using the Frymoyer (generalizable) model were within the range/mean SD of other studies. Comparison of simulated concentrations using the Frymoyer model against the observed/simulated concentrations of other studies is presented in Table 2. The recommended potential dosing regimen for individuals with BWT ranging from 2.5 to 4 kg and SCr values ranging from 0.2 to 1.5 mg/dL to maintain concentrations in the therapeutic range are presented in Table 3.

**Table 2:** Comparison of Frymoyer model simulated concentrations with other studies observed/simulated concentrations.

Study author	Mean/median doses as per studies	Peak concentration (mg/L)		Trough concentration (mg/L)	
		Reported	Simulated	Reported	Simulated
Cies, 2018	5 mg/kg for 36 h	10–12	10.8	<2	0.5
Bijleveld, 2016	5 mg/kg for 36 h	9.5 (7.5–11.9)	10.3	0.6 (0.3–0.8)	0.31
Ting, 2014	2.5 mg/kg for 12 h	7.57–12.71	8.3	2.29–5.52	3.2
Mark, 2013	4 mg/kg for 24 h	9.54 ± 1.30	9.54	1.68 ± 0.69	1.4
Frymoyer, 2013a	5 mg/kg 36 h	10.5 (7.8–13.5)	10.3	0.9 (0.3–2)	0.7
Frymoyer, 2013b	5 mg/kg for 36 h	10 ± 1.9	11.2	0.9 ± 0.4	0.9
Liu 2009	4 mg/kg for 24 h	>10	10.7	2.19 ± 1.7	2.3

Virtual subjects were generated for respective studies using their mean/median demographic details like birth weight, dose, dosing interval, and serum creatinine. The pharmacokinetic profile of these virtual subjects was simulated using Frymoyer model.^14^ Simulated concentrations were compared with the reported concentrations of respective studies.

**Table 3:** Dosage regimen recommendations to attain a therapeutic range of gentamicin.

	BWT (2.5–4 kg)
SCr	Gentamicin dosage of neonates based on per kg of birth weight
0.2–0.7 mg/dL	4 mg/kg Q 24 h
0.8–1.2 mg/dL	4 mg/kg Q 36 h
1.3–1.5 mg/dL	4 mg/kg Q 48 h

Simulations for these recommendations were carried out for the serum creatinine from the range 0.2–1.5 mg/dL with the intervals of 0.1 mg/dL. Birth weight ranges from 2.5 to 4 kg with intervals of 0.25 kg. These recommendations are expected to result in a peak concentration between 7.5 and 10 μg/mL and trough concentrations between 0.5 and 2 μg/mL.

## Discussion

Clinical trials in neonates are the most challenging in situations like PA and TH (PATH), leading to a paucity of data on drug PK in such conditions.^15^ PopPK modeling is a better way to conduct research in vulnerable populations.^16^ Without going through the typical clinical trial process, PopPK studies successfully described the PK of medications and proposed dosing regimens.^17^ Many PopPK studies have recommended dosage regimen based on their study population data, but generalizable dosage regimen recommendation that works on wider populations are very sparse. The meta-modeling method was reported to help in the identification of a generalizable model that can be used to provide dosing recommendations in a variety of populations.^18,19^

Reduced CL during TH has been reported for amikacin, amoxicillin, and ampicillin.^20–22^ Seven reports on gentamicin during PATH were used in the meta-modeling procedure of the present study. The majority of the reports on gentamicin showed a reduction in CL during TH, while there were reports of an increase and no change in CL as well.^14,23–28^ A recent meta-analysis reported that CL of gentamicin was reduced during TH.^29^ In this study, they have summarized the results from the published studies and reported reduced CL of gentamicin during TH. They focused on the combined impact of PA and TH. Since TH is PA’s only therapeutic option, it is difficult to investigate the fundamental reason of reduced CL. In the present study, we focused on the effects of PA and TH separately and the studies included in the present work^26,27^ had groups with and without TH during PA. In the present study, five reports^14,23–25,28^ compared the CL of neonates during PATH to the neonates without PA and TH. In these studies, the independent effect of PA or TH on CL could not be estimated as PA itself has been reported to cause renal impairment.^1,26,30,31^

Two studies compared neonates diagnosed with PA against neonates with PATH with conflicting findings.^26,27^ Mark et al. reported that neonates with PA had higher CL than neonates undergoing PATH.^27^ When looked at the demography, neonates in PATH group had lower GA and BWT, although it was not statistically significant. GA and BWT are critical for renal maturation^32^ and lower GA and BWT could have possibly contributed to reduced gentamicin CL. In another study, it was reported that there was no difference in CL between newborns with PA and PATH.^26^ There was no difference in terms of demographic characteristics like GA, BWT, and plasma creatinine in this study. According to the authors of this study, renal maturation is the key for CL of gentamicin, while TH has no effect on the CL.^26^

SCr is commonly used to evaluate renal function, but in asphyxiated infants, SCr may not accurately reflect renal function. It is crucial to keep track of the rate at which SCr drops in order to find out how well those newborns’ kidneys are functioning.^33^ As the rate of decline in SCr was not reported in any study, SCr measurement was the next best possibility to assess the renal function. To recommend gentamicin dose regimens to neonates with PA, it is important to consider GA, BWT, and SCr. Organ maturation can be explained by BWT, which is linearly connected to GA.^34,35^

One-compartment model with BWT and SCr as covariates on CL reported by Frymoyer et al. was successfully qualified by comparing with data from other studies.^14^ Adding SCr as a covariate in the model would help to understand the changes in renal function as gentamicin can induce renal toxicity even with shorter courses of high concentration dosage regimen.^36,37^ The Frymoyer et al. model was chosen as a generalizable model for two reasons: it successfully predicted concentrations reported in other papers and it has relevant covariates.

In two sets of population data, Sampson et al. validated the Frymoyer model’s predictive performance.^38^ In one dataset, it performed well, and in another one, it did not do well. In the group where the Frymoyer model did not perform well, GA was significantly lower than the other group and it included premature neonates. Frymoyer study did not include premature infants in its model development. Therefore, failed validation has different explanations and these have to be considered when evaluating the Frymoyer model. The authors also mentioned that the model performance of the failed validation improved when post-therapy samples were dropped.

In their study, Frymoyer et al.^14^ simulated doses ranging from 3 to 5 mg/kg at intervals of 24–48 h. Based on their simulation, the ideal dosing regimen was 4–5 mg/kg once every 36 h. To arrive at the optimal dosage regimen, we used the range of BWT and SCr values reported across all studies, as well as dosage intervals, ranged from 24 to 48 h. The current dosing recommendation is expected to reduce gentamicin trough exposure while maintaining optimal peak levels.

### Limitations

The current recommendations need to be clinically validated in a prospective study. Differences in estimation methods of gentamicin and SCr may impact model prediction ability. Many studies used neonates without PA as controls to assess the effect of TH on CL and this is not a prudent approach. In some studies, premature neonates were included in the control arm and the inferences from these studies have to be assessed carefully. None of these studies were randomized controlled study.

## Conclusion

For newborns undergoing PATH, available PopPK models of antimicrobial agents were collected. Gentamicin data were taken further for conducting a meta-model analysis. We identified the Frymoyer model as a generalizable model, which could match with data from other studies when simulations were carried out. BWT and SCr were found to be important covariates in this model. To maintain an appropriate therapeutic range, potential dosing regimens were recommended based on the value of covariates from the published studies. In infants receiving TH, the gentamicin dosage was suggested from the range of 4 mg/kg every 24 h to 4 mg/kg every 48 h. Prospective studies with these recommended regimens can aid in determining their effectiveness.

## Supplementary information

Supplementary Table
