# Supplementary material for: Pharmacometric approach to assist dosage regimen design in neonates undergoing therapeutic hypothermia
Source: Pediatr Res. 2021 Sep 7;92(1):249–54. doi: 10.1038/s41390-021-01714-0 (PMC9411058; doi:10.1038/s41390-021-01714-0)
Supplement: Supplementary file 1 — Supplementary Table [file 41390_2021_1714_MOESM1_ESM.docx]

Supplementary Table 1: Search Strategy

| **DATABASE** | **SEARCH STRATEGY** | **NO. OF ARTICLES** |
| --- | --- | --- |
| SCOPUS | (“infant*” OR “neonate*” OR “new born*”) AND (“asphyxia neonatorum*” OR “birth asphyxia*” OR “Perinatal asphyxia*” OR “hypoxic ischemic encephalopathy”) AND (“therapeutic hypothermia” OR “induced hypothermia” OR “controlled hypothermia” OR “cooling therapy”) AND (“Anti-Bacterial agent*” OR “Anti-Infective Agent*”) | 32 |
| PUBMED | (infant OR neonate OR new born) AND (asphyxia neonatorum OR birth asphyxia OR Perinatal asphyxia OR hypoxic ischemic encephalopathy) AND (therapeutic hypothermia OR induced hypothermia OR controlled hypothermia OR cooling therapy) AND (Anti-Bacterial agents OR Anti-Infective Agents) | 31 |
| OVID MEDLINE | (infant or neonate or new born) AND (asphyxia neonatorum or birth asphyxia or Perinatal asphyxia or hypoxic ischemic encephalopathy) AND (therapeutic hypothermia or induced hypothermia or controlled hypothermia or cooling therapy) AND (Anti-Bacterial agents or Anti-Infective Agents) | 4 |
| CINAHL | (infant OR neonate OR new born) AND (asphyxia neonatorum OR birth asphyxia OR Perinatal asphyxia OR hypoxic ischemic encephalopathy) AND (therapeutic hypothermia OR induced hypothermia OR controlled hypothermia OR cooling therapy) AND (Anti-Bacterial agents OR Anti-Infective Agents) | 8 |
